# Risk of thyroid dysfunction after COVID-19 vaccination: meta-analysis of 11 million individuals

**DOI:** 10.1097/MS9.0000000000004186

**Published:** 2025-10-27

**Authors:** Noheir Ashraf Ibrahem Fathy Hassan, Eman A. Toraih, Menna A. Shebl, Amaan Usmani, Amr Ossama Gaber Arafa, Mohamed Mohamed Elnouty, Ayesha Laraib-Ijaz, Uday Atwal, Deepak Gir, Helen A.O. Samuel, Mazen Hassanin, Nayiira Salim, Jaffer Shah, Yusef Hazimeh, Hani Aiash

**Affiliations:** a Faculty of Medicine, Aswan University, Aswan, Egypt; b Department of Cardiovascular Perfusion, College of Health Professions, Upstate Medical University, New York, NY, USA; c Genetics Unit, Histology and Cell Biology Department, Faculty of Medicine, Suez Canal University, Ismailia, Egypt; d Faculty of Medicine, Modern University for Technology and Information, Cairo, Egypt; e Skaneateles Central High School, Skaneateles, NY, USA; f Faculty of Medicine, Alexandria University, Alexandria, Egypt; g Faculty of Medicine, Cairo University, Cairo, Egypt; h Department of Internal Medicine, University Of Missouri, Kansas City, USA; i Fayetteville-Manlius High School, Manlius, NY, USA; j Department of Internal Medicine, St. Joseph’s Medical Center, Stockton, USA; k St. George University School of medicine, Grenada, West Indies; l Department of Ophthalmology, Weill Cornell Medicine, New York, NY, USA; m Department of Internal Medicine, Division of Endocrinology, American University of Beirut, Beirut, Lebanon

**Keywords:** COVID-19, Graves’ disease, thyroid, vaccination

## Abstract

**Background:**

To mitigate the SARS-CoV-2 pandemic, the worldwide rollout of COVID-19 vaccinations has been essential. Potential links between the COVID-19 vaccine and several thyroid disorders, like subacute thyroiditis, Graves’ disease, and thyroiditis, were shown by early case reports and short observational studies. The goal of this systematic review and meta-analysis is to assess the relationship between several COVID-19 vaccinations and thyroid conditions.

**Methods:**

We conducted a comprehensive search of major databases through February 2025, adhering to Preferred Reporting Items for Systematic Reviews and Meta-Analyses (PRISMA) criteria. Thyroid dysfunction after COVID-19 vaccination was the main outcome measure used in our analysis of 21 studies. Specific thyroid dysfunctions (Graves’ disease, hyperthyroidism, hypothyroidism, thyroiditis, and thyroid eye disease) were among the secondary outcomes. For every meta-analysis, we used random-effects models based on the Der Simonian and Laird approach, considering the anticipated clinical and methodological heterogeneity among trials.

**Results:**

Single-arm meta-analysis revealed thyroid dysfunction prevalence of 8.54% [95% confidence interval (CI) 3.45–19.61%] among vaccinated individuals compared to 31.78% (95% CI: 14.18–56.79%) in unvaccinated populations (*P* = 0.026) with significantly lower prevalence of Graves’ disease among vaccinated individuals (8.55 vs. 46.50%, *P* = 0.045; relative risk (RR) = 0.18, 95% CI: 0.04–0.92). Hyperthyroidism (1.11 vs. 1.55%, *P* = 0.90), hypothyroidism (1.13 vs. 5.13%, *P* = 0.44), and thyroiditis (0.26 vs. 3.90%, *P* = 0.49) showed no significant differences between vaccination groups; however, new-onset hyperthyroidism demonstrated significantly higher prevalence in vaccinated individuals (0.24 vs. 0.13%, *P* < 0.001; RR = 1.85, 95% CI: 1.84–1.86).

**Conclusion:**

This thorough meta-analysis offers comforting proof of the COVID-19 vaccines’ safety for the thyroid, with some protective benefits against Graves’ disease and mostly neutral relationships with other thyroid conditions.

## Introduction

The unprecedented global deployment of COVID-19 vaccines has been fundamental in mitigating the SARS-CoV-2 pandemic, with over 13 billion doses administered worldwide since December 2022^[1]^. These vaccines, developed across multiple platforms including mRNA, adenoviral vector, and inactivated virus technologies, have demonstrated strong overall safety profiles in clinical trials and post-marketing surveillance^[2,3]^. However, as vaccination campaigns have expanded, there has been growing interest in understanding potential associations between COVID-19 vaccines and autoimmune or inflammatory conditions, including thyroid dysfunction^[4]^.

Thyroid disorders represent some of the most common endocrine conditions worldwide, with varying prevalence rates across different populations. These disorders include hyperthyroidism, hypothyroidism, thyroiditis, Graves’ disease, and thyroid eye disease, among others^[5–9]^. The etiology of thyroid dysfunction is multifactorial, involving complex interactions between genetic predisposition, environmental factors, and immune system dysregulation^[5–9]^. Given the immunomodulatory effects of vaccines, several case reports and observational studies have examined potential temporal associations between COVID-19 vaccination and new-onset or relapse of thyroid disorders^[10]^.

The biological plausibility of vaccine-induced thyroid dysfunction is supported by several hypothesized mechanisms. These include molecular mimicry between vaccine components and thyroid antigens, bystander activation of autoreactive T-cells, immune complex formation, and general immune system activation following vaccination^[11–14]^. The spike protein of SARS-CoV-2, which is the immunogenic target for most COVID-19 vaccines, shares peptide sequences with various human proteins including some expressed in thyroid tissue, potentially triggering cross-reactive immune responses. Additionally, the robust immune activation following vaccination could theoretically precipitate autoimmune phenomena in susceptible individuals^[11–14]^.

Early case reports and small observational studies suggested potential associations between COVID-19 vaccination and various thyroid conditions, including subacute thyroiditis, Graves’ disease, and immune checkpoint inhibitor-like thyroiditis^[15–18]^. However, the clinical significance, frequency, and causal relationship of these associations remain poorly understood due to methodological limitations of individual studies, including small sample sizes, lack of appropriate control groups, and potential reporting and detection biases.

Despite the accumulating literature, there has been no comprehensive synthesis of evidence examining the relationship between COVID-19 vaccination and thyroid dysfunction across different populations, vaccine platforms, and specific thyroid disorders. Previous systematic reviews have primarily focused on case reports or limited outcomes, leaving substantial gaps in our understanding of this potential association^[19,20]^.

To address this knowledge gap, we conducted a meta-analysis of 21 studies with over 11 million patients across diverse regions, vaccine platforms, and study designs. Our objectives were to (1) estimate thyroid dysfunction frequency following COVID-19 vaccination compared to unvaccinated controls; (2) identify association patterns across different thyroid disorders, vaccine types, and population subgroups; and (3) evaluate the temporal relationship between vaccination and thyroid dysfunction onset.

This study aims to inform clinical management of thyroid function in vaccine recipients, guide vaccine safety policies, and direct future research priorities – especially important given vaccination’s role in pandemic control and the high prevalence of thyroid disorders globally.

## Methodology

### Study design

This systematic review and meta-analysis study follows the Preferred Reporting Items for Systematic Reviews and Meta-Analyses (PRISMA) guidelines. The protocol has been registered in PROSPERO (registration number: CRD42024586890).

**HIGHLIGHTS**

- This comprehensive meta-analysis examines the risk of various thyroid abnormalities following COVID 19 vaccination across 21 studies published between 2022 and 2025, comprising 11 276 514 patients (10 099 572 vaccinated individuals and 1 176 942 unvaccinated controls).
- Our research revealed thyroid dysfunction prevalence of 8.54% [95% confidence interval (CI): 3.45-19.61%] among vaccinated individuals compared to 31.78% (95% CI: 14.18–56.79%) in unvaccinated populations (*P* = 0.026) with significantly lower prevalence of Graves’ disease among vaccinated individuals (8.55 vs. 46.50%, *P* = 0.045; RR = 0.18, 95% CI: 0.04–0.92). Hyperthyroidism (1.11 vs. 1.55%, *P* = 0.90), hypothyroidism (1.13 vs. 5.13%, *P* = 0.44), and thyroiditis (0.26 vs. 3.90%, *P* = 0.49) showed no significant differences between vaccination groups; however, new-onset hyperthyroidism demonstrated significantly higher prevalence in vaccinated individuals (0.24 vs. 0.13%, *P* < 0.001; RR = 1.85, 95% CI: 1.84–1.86).
- Our manuscript makes several important contributions to the field. These findings support continued vaccination while highlighting the importance of personalized risk assessment, clinical vigilance for thyroid manifestations, and targeted research to enhance our understanding of immune-endocrine interactions following vaccination

### Eligibility criteria

We conducted a comprehensive literature search of electronic databases including PubMed, Medline, Web of Science, and Scopus, up to February 2025. The search strategy combined terms related to COVID-19 vaccination and thyroid dysfunction using the following keywords: “COVID-19” OR “SARS-CoV-2” OR “coronavirus” AND “vaccine” OR “vaccination” AND “thyroid” OR “hyperthyroidism” OR “hypothyroidism” OR “Graves’ disease” OR “thyroiditis” OR “thyroid eye disease.” Additional studies were identified through manual searching of reference lists and citation tracking of included articles.

### Eligibility criteria

Studies were included if they met the following criteria: (1) reported cases of thyroid dysfunction following COVID-19 vaccination; (2) included participants of any age; (3) provided information on the number of vaccinated and unvaccinated participants where applicable; (4) reported at least one thyroid dysfunction outcome (Graves’ disease, hyperthyroidism, hypothyroidism, thyroiditis, or thyroid eye disease); and (5) were published in English. We included observational studies (cohort, case control, and cross sectional), systematic reviews of reported cases, and clinical studies with relevant data.

Studies were excluded if they (1) were editorials, commentaries, or conference abstracts without sufficient clinical data; (2) did not provide adequate information about vaccination status; (3) focused exclusively on COVID-19 infection rather than vaccination; or (4) reported thyroid dysfunction without clear temporal relationship to vaccination.

### Data extraction

Data extraction was performed independently by two reviewers using a standardized form, with discrepancies resolved through discussion or consultation with a third reviewer. We extracted the following information: (1) study characteristics: First author, publication year, country, study design, study period, follow-up duration (mean and standard deviation where available), and types of vaccines examined; (2) population characteristics: Sample size (total, vaccinated, and unvaccinated), demographics (age means and standard deviations, sex distribution), and whether the study focused on new-onset cases or relapses of thyroid dysfunction; (3) thyroid dysfunction outcomes: Number of cases for each specific type of thyroid dysfunction (Graves’ disease, hyperthyroidism, hypothyroidism, thyroiditis, and thyroid eye disease) in both vaccinated and unvaccinated groups; and (4) vaccination details: Types of vaccines administered (mRNA, adenoviral, or inactivated), and vaccine-specific outcome data where available. Where studies reported multiple subgroups or various follow-up periods, we extracted data separately for each group to enable appropriate subgroup analyses.

### Quality assessment

We used the Newcastle-Ottawa Scale (NOS) for quality assessment of case control studies, and its adapted version for quality assessment of cross-sectional studies^[21,22]^. Case-control studies were assessed for three domains: selection of study groups (0–4 points), comparability of groups (0–2 points), and ascertainment of exposure or outcome (0–3 points), while cross-sectional studies (maximum 9 points) were assessed for selection (sample size, representativeness, tool validation, and response rate; 0–4 points), comparability (group matching, confounder analysis; 0–2 points), and exposure (outcome assessment, statistical methods; 0–3 points). For systematic reviews of reported cases, we employed the Joanna Briggs Institute (JBI) Critical Appraisal Checklist.

Two reviewers independently conducted quality assessments, with disagreements resolved through discussion or consultation with a third reviewer. Studies were categorized as high quality (NOS ≥ 7 or equivalent JBI scores), moderate quality (NOS 5–6), or low quality (NOS ≤ 4).

### Statistical analysis

The primary outcome measure was the occurrence of any thyroid dysfunction following COVID-19 vaccination. Secondary outcomes included specific types of thyroid dysfunction (Graves’ disease, hyperthyroidism, hypothyroidism, thyroiditis, and thyroid eye disease). For studies with appropriate control groups, we calculated relative risk (RR) with 95% confidence intervals (CI) comparing the occurrence of thyroid dysfunction between vaccinated and unvaccinated individuals.

Given the expected clinical and methodological heterogeneity across studies, we employed random-effects models using the DerSimonian and Laird method for all meta-analyses. Heterogeneity among studies was assessed using the *I*^2^ statistic, with values of 25, 50, and 75% considered as low, moderate, and high heterogeneity, respectively. The χ^2^ test was used to evaluate the statistical significance of heterogeneity.

We conducted pre-specified subgroup analyses based on type of thyroid dysfunction (Graves’ disease, hyperthyroidism, hypothyroidism, thyroiditis, and thyroid eye disease), vaccine platform (mRNA, adenoviral, and inactivated), study design (cohort, case control, and cross sectional), geographic region, and disease onset pattern of thyroid dysfunction (new-onset vs. relapse).

Sensitivity analyses were conducted to test the robustness of pooled estimates by excluding studies with lower methodological quality. Baujat plots were used to identify studies contributing most to heterogeneity and effect size influence. Publication bias was evaluated using funnel plots and Egger’s test when at least 10 studies were available for a specific outcome. All statistical analyses were conducted using R version 4.2.0 (R Foundation for Statistical Computing, Vienna, Austria) with the “meta” package.

## Results

### Study selection and characteristics

**Figure 1.**
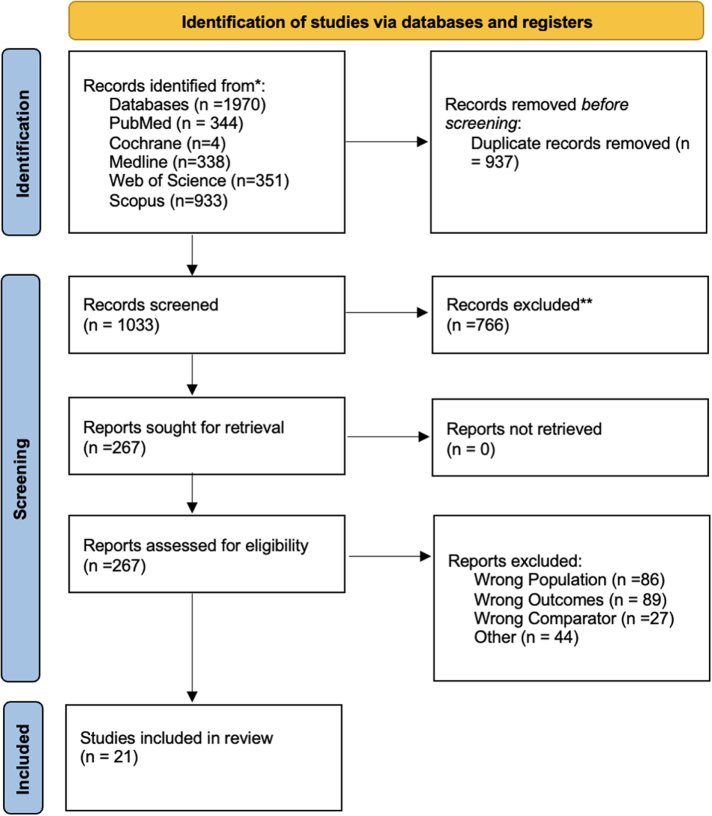
Preferred Reporting Items for Systematic Reviews and Meta-Analyses flow diagram of study selection process.

Our systematic search identified 21 eligible studies with 22 datasets examining thyroid dysfunction following COVID-19 vaccination (Fig. 1). These studies collectively included 11 276 514 patients (10 099 572 vaccinated individuals and 1 176 942 unvaccinated controls). Studies were published between 2022 and 2025, with geographic distribution across Asia (*n* = 8), Europe (*n* = 8), the Middle East (*n* = 3), and the Americas (*n* = 2). The methodological profile included retrospective designs (*n* = 13), prospective cohorts (*n* = 5), cross-sectional studies (*n* = 2), and case series (*n* = 2). Sample sizes varied substantially, ranging from 27 participants^[23]^ to over 5.4 million^[24]^. Table 1 presents the complete characteristics of included studies.

**Table 1:** Characteristics of included studies

Author,year	Country	Study design	Study period	Vaccine type(s)	Subgroup	Total sample size	Mean age (SD)	Male (%)	Ref
Cheng, 2025	Taiwan	Retrospective	12 months	mRNA and Adenoviral	New onset	2 333 496	43.72 (25.69)	44.9	^[25]^
Pollack-Schreiber, 2024	USA	Retrospective	09/2017–08/2022	—	New onset	156	12.5 (4.0)	—	^[26]^
Muller, 2024	Italy	Case series	01/2021–08/2022	—	New onset	81	50.8 (15.6)	30.9	^[27]^
Duskin-Bitan, 2024	Israel	Retrospective	—	—	New onset	12 895	—	—	^[28]^
Bea, 2024	South Korea	Case series	02/2021–12/2021	mRNA and Adenoviral	New onset	5 407 214	—	—	^[24]^
					Relapse	31 783	—	—	
Xu, 2023	China	Prospective	06/2021–09/2021	—	Relapse	601	43.76 (16.68)	18.8	^[29]^
Wong, 2023	China	Prospective	01/2021–09/2021	mRNA and Inactivated	Relapse	342	51.6 (16.1)	—	^[30]^
Sezgin, 2023	Turkey	Cross sectional	06/2021–08/2021	mRNA and Inactivated	New onset	314	37.62 (10.7)	52.2	^[31]^
Natal, 2023	Brazil	Retrospective	01/2019–05/2022	—	New onset	7531	45.29 (13.55)	—	^[32]^
Herczeg, 2023	Hungary	Prospective	03/2021–03/2022	mRNA	New onset	452	12.4 (3.8)	—	^[33]^
Gorshtein, 2023	Israel	Retrospective	12/2020–11/2022	mRNA	New onset	2178	—	—	^[34]^
Galindo, 2023	Spain	Cross sectional	2017–2021	mRNA and Adenoviral	New onset	66	48.9 (15.6)	—	^[35]^
Endo, 2023	USA	Retrospective	12/2017–10/2022	mRNA, Adenoviral, and Inactivated	New onset	121	43.73 (15.63)	—	^[36]^
Batman, 2023	Turkey	Retrospective	01/2022–04/2022	mRNA and Inactivated	New onset	811	—	—	^[37]^
Zhao, 2022	China	Retrospective	09/2021–01/2022	Inactivated	New onset	6979	—	—	^[38]^
Wong, 2022	China	Retrospective	02/2021–09/2021	mRNA and Inactivated	New onset	2 288 239	—	—	^[39]^
Tekin, 2022	Turkey	Retrospective	03/2019–12/2021	mRNA and Inactivated	New onset	27	44.77 (10.58)	37.0	^[23]^
Lui, 2022	China	Prospective	06/2021–08/2021	mRNA and Inactivated	New onset	215	49.6	37.2	^[40]^
Li, 2022	China	RCT and prospective	04/2021–06/2021	Inactivated	New onset	564	32.8 (9.2)	—	^[41]^
Garcia, 2022	Spain	Retrospective	—	mRNA, Adenoviral, and Inactivated	New onset	1 182 394	—	—	^[42]^
di Fillippo, 2022	Italy	Retrospective	01/2021–12/2021	mRNA and Adenoviral	New onset	55	40 (17.7)	25.5	^[43]^

Dashes (—) indicate data not available or not reported in the original study; RCT: randomized controlled trial.

Nineteen datasets examined new-onset thyroid dysfunction, while three focused on relapse of pre-existing thyroid conditions. Regarding vaccine platforms, 13 studies evaluated multiple vaccine types (primarily mRNA with other vaccines), 3 examined exclusively mRNA vaccines, and 3 investigated inactivated vaccines.

## Quality assessment

The methodological quality of included studies was evaluated using a modified NOS. Among cohort and case-control studies (*n* = 19), 31.5% achieved high-quality scores (8–9 points) and 52.5% medium quality (6–7 points), Supplemental Digital Content Table S1, available at: http://links.lww.com/MS9/B6. Cross-sectional studies (*n* = 2) showed medium quality (5 points), Supplemental Digital Content Table S2, available at: http://links.lww.com/MS9/B6.

### Demographic characteristics

**Figure 2.**
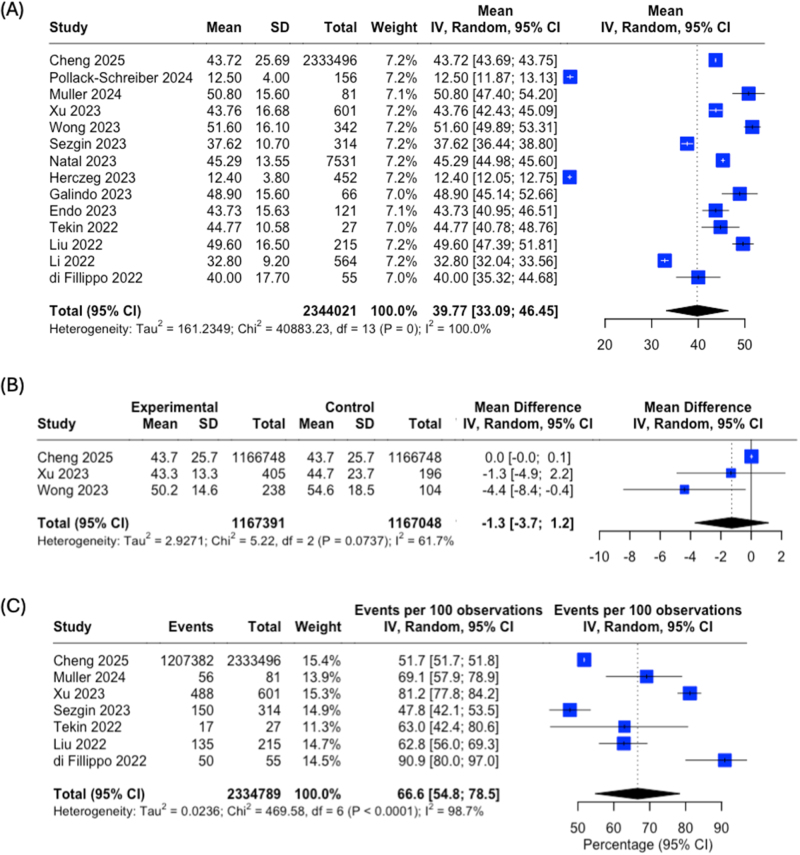
**Demographic characteristics of study participants.** (A) Forest plot showing mean age of participants across 14 studies with available age data. (B) Forest plot comparing mean age between vaccinated (experimental) and unvaccinated (control) groups from three studies with available data. (C) Forest plot of female representation across seven studies reporting gender distribution. Squares represent study-specific estimates with size proportional to study weight; horizontal lines indicate 95% confidence intervals; diamonds represent pooled estimates with width indicating 95% confidence intervals.

The mean age of participants ranged from 12.4 years^[33]^ to 51.6 years^[30]^, with a pooled mean of 39.8 years (95% CI: 33.1–46.5; Fig. 2A). No significant age difference was observed between vaccinated and unvaccinated groups (mean difference −1.3, 95% CI: −3.7 to 1.2; Fig. 2B). Gender distribution was reported in seven studies, with female predominance across studies (pooled percentage 66.6%, 95% CI: 54.8–78.5%; Fig. 2C).

## Overall thyroid dysfunction analysis

**Figure 3.**
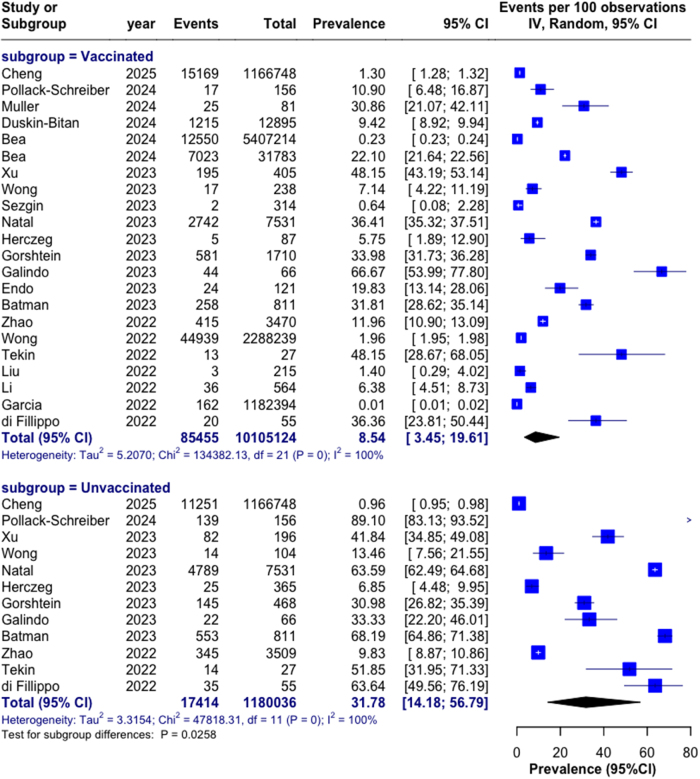
**Prevalence and relative risk of thyroid dysfunction.** Forest plot showing the prevalence of thyroid dysfunction in vaccinated and unvaccinated populations. Squares represent study-specific estimates with size proportional to study weight; horizontal lines indicate 95% confidence intervals; diamonds represent pooled estimates with width indicating 95% confidence intervals.

Single-arm meta-analysis revealed thyroid dysfunction prevalence of 8.54% (95% CI: 3.45–19.61%) among vaccinated individuals compared to 31.78% (95% CI: 14.18–56.79%) in unvaccinated populations (*P* = 0.026; Fig. 3). Substantial heterogeneity was observed (*I*^2^ = 100%). Comparative analysis demonstrated a significant protective association with vaccination across all datasets (RR = 0.27, 95% CI: 0.14–0.51, *P* < 0.001; Table 2).

**Table 2:** Overall thyroid dysfunction risk following COVID-19 vaccination

Subgroup	Studies (*k*)	Vaccinated prevalence (95% CI)	Unvaccinated prevalence (95% CI)	Relative risk (95% CI)	*p*-value
All studies	22	8.54% (3.45–19.61)	31.78% (14.18–56.79)	0.27 (0.14–0.51)	**<0.001**
Geographic region					
Asia	8	1.69% (0.31–8.67)	2.48% (0.89–6.71)	0.68 (0.46–1.01)	0.06
Americas	3	22.13% (8.74–46.05)	63.59% (52.49–73.56)	0.35 (0.14–0.87)	**0.023**
Europe	5	36.10% (1.45–73.62)	35.11% (15.44–61.52)	1.03 (0.44–2.41)	0.94
Middle East and North Africa	6	9.92% (0.93–56.46)	32.15% (8.55–70.51)	0.31 (0.12–0.78)	**0.013**
Study design					
Retrospective	12	4.76% (1.02–19.52)	19.48% (5.73–48.97)	0.24 (0.12–0.50)	**<0.001**
Prospective	5	11.05% (1.51–48.94)	24.45% (10.42–47.41)	0.45 (0.23–0.88)	**0.020**
Case series	3	0.63% (0.05–7.44)	—	—	—
Cross sectional	2	14.92% (0.19–95.13)	33.33% (22.20–46.01)	0.45 (0.07–2.85)	0.39
Onset pattern					
New onset	19	8.54% (3.45–19.61)	31.78% (14.18–56.79)	0.27 (0.14–0.51)	**<0.001**
Relapse	3	16.89% (3.84–50.54)	24.82% (9.74–50.15)	0.68 (0.46–1.01)	0.05

Prevalence values are presented as percentages with 95% confidence intervals in parentheses. Relative risks compare thyroid dysfunction risk in vaccinated versus unvaccinated individuals. *P*-values represent tests for subgroup differences. Bolded P-values indicate statistical significane (*P* < 0.05). Dashes (—) indicate insufficient data for calculation.

Stratification by geographic region, study design, and onset pattern revealed important effect modifiers (Table 2). Protective associations were strongest in studies from the Americas (RR = 0.35, *P* = 0.023) and Middle East/North Africa (RR = 0.31, *P* = 0.013), with no significant effect in Asian studies (*P* = 0.058) and European studies (*P* = 0.94). Regarding study methodology, stronger protective associations were observed in retrospective (RR = 0.24, *P* < 0.001) and prospective studies (RR = 0.45, *P* = 0.020) compared to cross-sectional designs (*P* = 0.39). Disease history significantly influenced outcomes, with new-onset thyroid dysfunction showing more pronounced protective association (RR = 0.27, *P* < 0.001) than relapse cases (*P* = 0.053).

### Thyroid dysfunction types

Analysis by specific thyroid disorder revealed differential associations with vaccination (Table 3). Graves’ disease showed significantly lower prevalence among vaccinated individuals (8.55% vs. 46.50%, *P* = 0.045; RR = 0.18, 95% CI: 0.04–0.92). This protective effect was consistent across both new-onset cases (8.91% vs. 57.03%, *P* = 0.021; RR = 0.16, 95% CI: 0.02–0.97) and relapse cases (7.95% vs. 13.46%, *P* = 0.05; RR = 0.59, 95% CI: 0.33–0.96).

**Table 3:** Subgroup analysis by specific thyroid dysfunction type

Stratification	Subgroup	Studies (*k*)	Vaccinated prevalence (95% CI)	Unvaccinated prevalence (95% CI)	Relative risk (95% CI)	*P*-value
Graves’ disease						
*Overall*	All studies	7/5	8.55 (1.17–42.56)	46.50 (18.76–76.5)	0.18 (0.04–0.92)	**0.045**
*By region*	Asia	3/1	0.03 (0.0–0.27)	13.46 (8.14–21.46)	0.06 (0.01–0.33)	**0.001**
	Americas	2/1	67.5 (7.85–98.03)	89.10 (83.13–93.52)	0.76 (0.51–1.13)	0.17
	Europe/MENA	2/3	52.4 (15.8–86.51)	50.18 (28.43–71.82)	1.05 (0.61–1.80)	0.87
*By onset pattern*	New onset	5/4	8.91 (0.49–66.14)	57.03 (26.00–83.37)	0.16 (0.02–0.97)	**0.021**
	Relapse	2/1	7.95 (6.09–10.32)	13.46 (8.14–21.46)	0.59 (0.33–0.96)	**0.050**
*By study design*	Retrospective	4/3	4.94 (0.07–80.3)	40.18 (17.05–68.5)	0.12 (0.01–1.61)	0.11
	Prospective	2/1	7.95 (6.09–10.3)	13.46 (8.14–21.46)	0.59 (0.33–0.96)	**0.050**
	Cross sectional	1/1	66.0 (53.9–77.8)	33.33 (22.2–46.01)	2.00 (1.37–2.93)	**<0.001**
Hyperthyroidism						
*Overall*	All studies	6/2	1.11 (0.15–7.89)	1.55 (0.01–66.88)	0.72 (0.01–41.3)	0.90
*By region*	Asia	5/2	0.94 (0.09–9.08)	1.55 (0.01–66.88)	0.61 (0.01–36.1)	0.82
	MENA	1/0	0.32 (0.08–1.26)	—	—	—
*By onset pattern*	New onset	4/1	0.24 (0.07–0.84)	0.13 (0.13–0.14)	1.85 (1.84–1.86)	**<0.001**
	Relapse	2/1	18.32 (13.4–24.53)	15.82 (11.35–21.6)	1.16 (0.74–1.80)	0.52
*By study design*	Retrospective	2/1	0.14% (0.13–0.15)	0.13 (0.13–0.14)	1.06 (1.05–1.07)	**<0.001**
	Prospective/case series	3/1	8.78 (0.48–66.4)	15.8 (11.35–21.6)	0.56 (0.09–3.37)	0.52
	Cross sectional	1/0	0.32 (0.08–1.26)	—	—	—
Hypothyroidism						
*Overall*	All studies	6/2	1.13 (0.25–4.94)	5.13 (0.14–67.84)	0.22 (0.01–5.30)	0.44
*By region*	Asia	5/2	1.12 (0.23–5.32)	5.13 (0.14–67.84)	0.22 (0.01–5.57)	0.45
	MENA	1/0	0.32 (0.08–1.26)	—	—	—
*By onset pattern*	New onset	4/1	0.48 (0.17–1.38)	0.83 (0.81–0.85)	0.58 (0.20–1.67)	0.31
	Relapse	2/1	5.80 (0.24–61.34)	26.02 (20.36–32.61)	0.22 (0.01–3.73)	0.29
*By study design*	Retrospective	2/1	0.84 (0.82–0.85)	0.83 (0.81–0.85)	1.01 (0.98–1.04)	0.47
	Prospective/case series	3/1	1.63 (0.08–27.25)	26.02 (20.36–32.61)	0.06 (0.00–1.03)	0.053
	Cross sectional	1/0	0.32 (0.08–1.26)	—	—	—
Thyroiditis						
*Overall*	All studies	7/3	0.26 (0.01–7.58)	3.90 (0.00–98.03)	0.07 (0.00–15.62)	0.49
*By region*	Asia	3/1	0.02 (0.0–0.70)	0.03 (0.02–0.04)	0.60 (0.02–22.04)	0.78
	Europe/MENA	4/2	12.85 (0.37–85.77)	51.85 (31.95–71.33)	0.25 (0.01–4.78)	0.35
*By study design*	Case series	2/0	0.66 (0.0–96.17)	—	—	—
Eye disease						
*Overall*	All studies	2/0	0.66 (0.0–96.17)	—	—	—
*By region*	Asia	1/0	0.01 (0.01–0.01)	—	—	—
	Europe	1/0	30.86 (2.11–42.11)	—	—	—
*By study design*	Case series	2/0	0.66 (0.0–96.17)	—	—	—

MENA, Middle East and North Africa.

Study counts are presented as vaccinated/unvaccinated. Prevalence values are presented as percentages with 95% confidence intervals in parentheses. Relative risks compare thyroid dysfunction risk in vaccinated versus unvaccinated individuals. *P*-values represent tests for subgroup differences. Dashes (–) indicate insufficient data for calculation.

In contrast, other thyroid conditions showed variable outcomes. Hyperthyroidism overall showed no significant difference between groups (1.11% vs. 1.55%, *P* = 0.90), though new-onset hyperthyroidism demonstrated significantly higher prevalence in vaccinated individuals (0.24% vs. 0.13%, *P* < 0.001; RR = 1.85, 95% CI: 1.84–1.86). Hypothyroidism (1.13% vs. 5.13%, *P* = 0.44) and thyroiditis (0.26% vs. 3.90%, *P* = 0.49) showed no significant differences between vaccination groups.

Further stratification of thyroid dysfunction subtypes by region, study design, and disease onset revealed additional patterns (Table 3). For Graves’ disease, the protective effect of vaccination was most pronounced in Asian studies (RR = 0.06, *P* = 0.001) compared to the Americas (RR = 0.76, *P* = 0.17) and Europe/MENA (RR = 1.05, *P* = 0.87). Study design also influenced findings, with cross-sectional studies showing significantly increased risk (RR = 2.0, *P* < 0.001) compared to the protective associations observed in prospective studies (RR = 0.59, *P* = 0.050).

### Vaccine platform analysis

**Figure 4.**
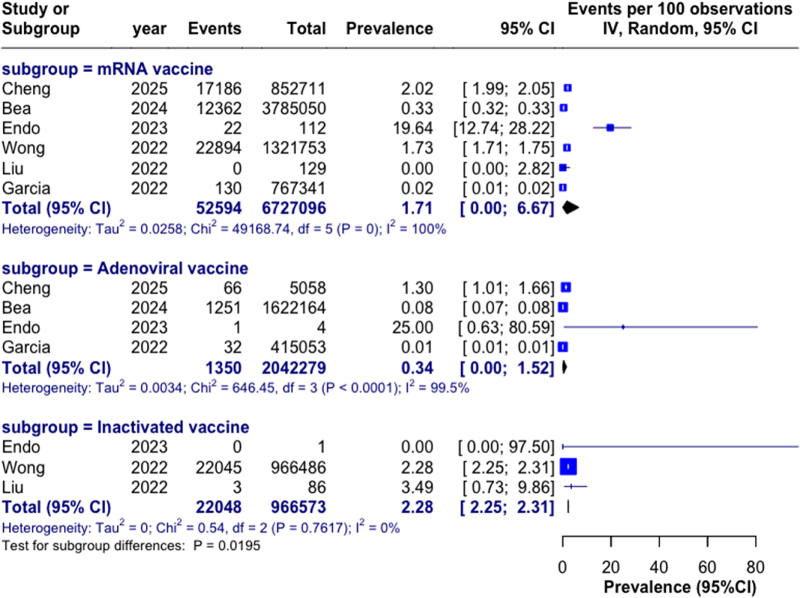
**Prevalence of thyroid dysfunction by vaccine type.** Forest plot showing prevalence of thyroid dysfunction stratified by vaccine platform: mRNA vaccines (*n* =6 studies), adenoviral vector vaccines (*n* =4 studies), and inactivated vaccines (*n* =3 studies). Squares represent study-specific estimates with size proportional to study weight; horizontal lines indicate 95% confidence intervals; diamonds represent pooled estimates with width indicating 95% confidence intervals. Test for subgroup differences: *P* = 0.019.

Significant differences in thyroid dysfunction prevalence were observed across vaccine platforms (*P* = 0.019; Fig. 4). Inactivated vaccines were associated with highest prevalence (2.28%, 95% CI: 2.25–2.31%), followed by mRNA vaccines (1.71%, 95% CI: 0.0–6.67%), and adenoviral vaccines (0.34%, 95% CI: 0.0–1.52%). The significant difference was primarily driven by lower rates with adenoviral compared to inactivated vaccines (*P* = 0.008), while no significant differences were observed between mRNA and other platforms.

## Sensitivity analysis

**Figure 5.**
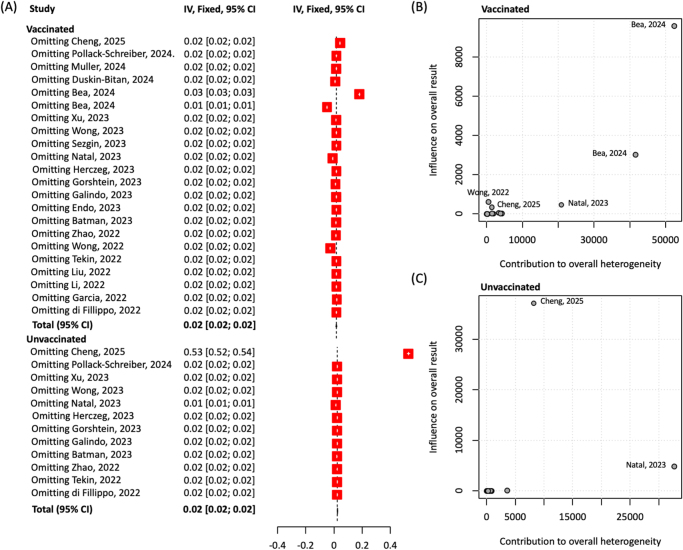
**Sensitivity analysis and study influence plots.** (A) The leave-one-out sensitivity analysis, where each study is sequentially omitted to assess its influence on the pooled effect estimate. The overall effect remains stable at 0.02 (95% CI: 0.02–0.02) across iterations, indicating robustness. However, four studies – Bea (2024), Cheng (2025), Wong (2022), and Zhao (2023) – show slight deviations in effect size or confidence intervals, suggesting modest influence on the pooled result. (B) Baujat plot restricted to the vaccinated group, where Bea (2024) exerts the greatest influence on both effect size and heterogeneity. (C) The same analysis for the unvaccinated group, identifying Cheng (2025) as the most influential study in that subgroup.

Leave-one-out sensitivity analysis showed that the overall effect estimate remained stable at 0.02 (95% CI: 0.02–0.02), indicating robustness to the exclusion of individual studies. Minor deviations were observed when omitting Bea (2024), Cheng (2025), Wong (2022), and Zhao (2023), suggesting modest influence. Subgroup analyses identified Bea (2024) and Cheng (2025) as the most influential in the vaccinated and unvaccinated groups, respectively (Fig. 5).

## Meta-regression analysis

Meta-regression analysis was conducted to elucidate sources of heterogeneity between studies, identifying significant moderators of the association between COVID-19 vaccination and thyroid dysfunction. Our model explained 62.8% of between-study variance in thyroid dysfunction risk (Table 4). Geographic region emerged as a significant effect modifier, with studies from the Americas (coefficient = −0.68, *P* = 0.023) and Middle East/North Africa (coefficient = −0.79, *P* = 0.014) demonstrating more pronounced protective associations compared to Asian studies. Study design also influenced effect estimates, with prospective studies showing less protective effect than retrospective studies (coefficient = 0.61, *P* = 0.021). Vaccination appeared more effective at preventing new-onset thyroid dysfunction than preventing recurrence in patients with history of thyroid disease (coefficient = −0.93, *P* < 0.001).

**Table 4:** Meta-regression analysis of factors influencing effect sizes

Moderator variable	Coefficient	95% CI	*P*-value	*R*^2^
**Model 1:** Overall thyroid dysfunction				
Intercept	0.24	−1.13; 1.61	0.73	62.8%
Geographic region (ref: Asia)				
Americas	−0.68	−1.27; −0.09	0.023	
Europe	0.42	−0.16; 1.00	0.16	
MENA	−0.79	−1.42; −0.16	0.014	
Study design (ref: Retrospective)				
Prospective	0.61	0.09; 1.13	0.021	
Case series	0.31	−0.53; 1.15	0.47	
Cross sectional	0.68	−0.27; 1.63	0.16	
Onset pattern (ref: relapse)				
New onset	−0.93	−1.44; −0.42	<0.001	
Publication year (continuous)	−0.24	−0.62; 0.14	0.21	
**Model 2:** Graves’ disease only				
Intercept	−0.97	−2.54; 0.60	0.22	83.5%
Geographic region (ref: Asia)				
Americas	2.53	1.63; 3.43	<0.001	
Europe/MENA	2.87	2.02; 3.72	<0.001	
Study design (ref: retrospective)				
Prospective	1.67	0.68; 2.66	0.001	
Cross sectional	2.84	1.64; 4.04	<0.001	
Onset pattern (ref: relapse)				
New onset	−1.31	−2.14; −0.48	0.002	
**Model 3:** Analysis by vaccine type				
Intercept	0.82	−0.07; 1.71	0.07	57.4%
Vaccine type (ref: inactivated)				
mRNA	−0.28	−0.77; 0.21	0.26	
Adenoviral	−1.93	−3.35; −0.51	0.008	

MENA, Middle East and North Africa; Ref, reference category.

Meta-regression analyses examined the influence of geographic region, study design, disease onset pattern, publication year, and vaccine platform on effect estimates. Meta-regression coefficients represent the change in log relative risk associated with each moderator variable. Negative coefficients indicate lower relative risk (more protective effect). *R*^2^ represents the proportion of between-study variance explained by moderators.

For Graves’ disease specifically, meta-regression explained 83.5% of heterogeneity, with pronounced regional effects (Americas: coefficient = 2.53, *P*<0.001; Europe/MENA: coefficient = 2.87, *P* < 0.001 vs. Asian studies). Regarding vaccine platforms, adenoviral vector vaccines were associated with significantly reduced risk compared to inactivated vaccines (coefficient = −1.93, *P* = 0.008), supporting our earlier subgroup findings.

## Publication bias assessment

**Figure 6.**
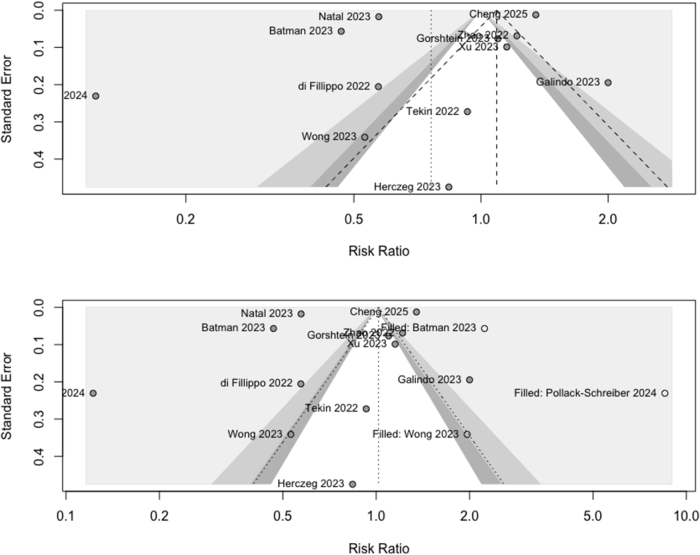
Publication bias assessment of studies examining thyroid dysfunction following COVID-19 vaccination. Funnel plot displays log risk ratios (x-axis) against standard error (y-axis) for each included study. Open circles represent observed studies; filled circles indicate potentially missing studies identified through trim-and-fill analysis. The vertical line represents the pooled effect estimate (RR = 0.76, 95% CI: 0.54–1.08), while the adjusted estimate after trim-and-fill correction was RR = 1.02 (95% CI: 0.74–1.40). The relative symmetry and statistical tests (Egger’s test: *P* = 0.516; Begg’s test: *P* = 0.284) suggest minimal publication bias despite substantial heterogeneity between studies (*I*^2^ = 99.4%).

Egger’s linear regression test (*t* = −0.67, df = 10, *P* = 0.52) and Begg’s rank correlation test (τ = −0.24, *P* = 0.28) indicated no significant publication bias (Fig. 6). Trim-and-fill analysis identified three potentially missing studies, with the adjusted random effects model shifting from RR = 0.76 (95% CI: 0.54–1.08) to RR = 1.02 (95% CI: 0.74–1.40). While this adjustment changed the point estimate direction, both CIs included the null value, suggesting no significant association either before or after adjustment. The persistent heterogeneity (*I*^2^ = 99.4%) suggested that genuine differences in study characteristics, rather than publication bias alone, explained the observed variation in effect estimates.

## Discussion

This meta-analysis of 21 studies encompassing over 11 million individuals represents the most comprehensive evaluation to date of thyroid dysfunction following COVID-19 vaccination, providing unprecedented statistical power to challenge earlier safety concerns from smaller studies. Our findings reveal a nuanced relationship characterized by significant heterogeneity across thyroid disorder types, geographic regions, vaccine platforms, and study methodologies, moving beyond the limited scope of previous case reports to provide robust epidemiological evidence. The most striking finding is the significant protective association between COVID-19 vaccination and Graves’ disease, particularly pronounced in Asian populations. This protection was observed for both new-onset and relapse cases, suggesting that vaccination may modulate autoimmune thyroid responses across different disease stages. In contrast, no significant associations were observed for hypothyroidism or thyroiditis, while new-onset hyperthyroidism showed increased prevalence in vaccinated individuals.

Our findings directly challenge initial concerns raised by early case reports suggesting increased thyroid autoimmunity risk following COVID-19 vaccination^[19,20,44,45]^. While previous smaller studies suggested vaccination might trigger autoimmune thyroid disorders^[10,16,46]^, our comprehensive analysis reveals the opposite – a significant protective association with Graves’ disease (RR = 0.18, 95% CI: 0.04–0.92). This contradiction likely reflects publication bias in early literature favoring unusual or adverse outcomes, whereas our comprehensive analysis captures the broader epidemiological landscape and provides clinicians with confidence when counseling patients. Unlike previous systematic reviews that primarily focused on case reports or limited outcomes^[19,20]^, our study provides the first large-scale quantitative synthesis across multiple thyroid disorders, vaccine platforms, and geographic regions, enabling identification of previously unrecognized patterns including significant regional variations and vaccine-specific differences with important clinical implications.

The substantial heterogeneity observed across studies (*I*^2^ ~ 100%) represents a critical limitation requiring careful interpretation of pooled estimates. This heterogeneity reflects significant differences among research populations, methodologies, geographical areas, vaccination platforms, and outcome criteria. Sources include methodological diversity ranging from retrospective database analyses to prospective clinical trials, population heterogeneity with varying baseline thyroid disease prevalence and genetic susceptibilities across regions, outcome definition variability affecting case ascertainment, and temporal factors covering different pandemic periods with varying vaccination schedules. While meta-regression and subgroup analyses explained 62.8–83.5% of between-study variance, unmeasured confounders and residual heterogeneity likely persist, necessitating cautious interpretation of results that may not be completely generalizable across all populations and clinical contexts. Paradoxically, this heterogeneity strengthens our findings by demonstrating consistency of the protective effect against Graves’ disease across diverse settings, lending credibility to this unexpected finding.

Our meta-regression analyses identified several significant moderators of vaccination effects that were previously unrecognized. Geographic region emerged as particularly important, with stronger protective associations in the Americas and Middle East/North Africa for overall thyroid dysfunction, yet stronger protection against Graves’ disease specifically in Asian populations. The pronounced regional variations likely reflect multiple factors including higher baseline Graves’ disease prevalence and different HLA haplotype distributions (particularly HLA-B*46:01, HLA-DRB1*08:03, and HLA-DPB1*05:01) in Asian populations^[47,48]^, dietary patterns with higher iodine intake from seafood consumption in East Asian countries potentially modifying vaccination effects^[49–51]^, and more systematic thyroid screening programs in Asian health care systems enhancing detection of subtle parameter changes^[52–55]^. Study methodology significantly influenced findings, with retrospective and prospective studies showing stronger protective effects than cross-sectional designs. Vaccine platform also proved important, with adenoviral vaccines associated with lower thyroid dysfunction rates compared to inactivated vaccines, likely reflecting distinct immunological mechanisms where adenoviral vectors primarily induce strong cellular immune responses while inactivated vaccines predominantly elicit humoral immunity^[56–58]^.

Several biological mechanisms could explain the observed protective association with Graves’ disease. COVID-19 vaccination might enhance regulatory T-cell responses that attenuate autoimmune processes^[59]^, vaccine-induced interferon signaling could temporarily suppress autoreactive B-cells involved in thyroid autoantibody production^[60]^, and vaccine-induced antibodies might competitively bind to targets that would otherwise interact with pathogenic autoantibodies affecting thyroid tissue^[61]^. Conversely, the increased risk of new-onset hyperthyroidism in vaccinated individuals likely reflects subclinical thyroiditis triggered by vaccine-induced inflammatory responses^[62,63]^, aligning with documented cases of subacute thyroiditis following vaccination that typically manifests as transient hyperthyroidism followed by recovery or hypothyroidism^[15,64–66]^. The differential effects by thyroid disorder subtype suggest that vaccination influences distinct immunopathological pathways rather than exerting uniform effects on thyroid function.

From a clinical standpoint, our findings offer several key evidence-based learning points for health care providers. Clinicians can confidently reassure patients with existing thyroid conditions that vaccination is safe and potentially beneficial, directly addressing widespread concerns about autoimmune complications that have contributed to vaccine hesitancy. However, they should remain vigilant for hyperthyroid symptoms including palpitations, tremor, heat intolerance, and weight loss in the weeks following vaccination, while understanding these tend to be mild and transient. The differential effects across thyroid disorder types indicate that routine post-vaccination thyroid function screening is not necessary for the general population, though symptom-based evaluation remains appropriate particularly in patients with pre-existing thyroid conditions or risk factors. The regional variations in vaccine-thyroid associations highlight the importance of incorporating demographic and geographic factors into personalized risk assessment, with Asian patients potentially deriving greater protection against Graves’ disease. The observed differences across vaccine platforms, particularly lower thyroid dysfunction rates with adenoviral vaccines, may inform selection discussions for high-risk individuals, though this should be balanced against overall vaccine efficacy and availability.

Our analysis has several strengths including comprehensive scope, unprecedented sample size exceeding 11 million individuals, rigorous methodology with extensive subgroup and meta-regression analyses, inclusion of diverse study designs and geographic regions enhancing generalizability, and publication bias assessment confirming robustness of findings. Important limitations warrant acknowledgment including the observational nature of most included studies with inherent risks of confounding and selection bias, variability in thyroid dysfunction definitions and diagnostic criteria potentially influencing outcome ascertainment, inconsistently reported temporal relationships between vaccination and thyroid dysfunction limiting causal inference, and potential inability to capture rare adverse events requiring larger populations or longer follow-up than currently available. Given the substantial heterogeneity observed, future research should prioritize standardized approaches including uniform diagnostic criteria, standardized follow-up periods, inclusion of genetic and environmental modifying factors, and longer-term studies to assess persistence of observed effects.

## Conclusion

This comprehensive meta-analysis provides reassuring evidence regarding thyroid safety of COVID-19 vaccines, with potential protective effects against Graves’ disease and largely neutral associations with other thyroid disorders. The significant heterogeneity observed across studies reflects the complex, multifactorial nature of vaccine-thyroid interactions influenced by genetic, environmental, methodological, and immunological factors, yet the consistency of the protective effect across diverse populations strengthens these findings. These results support continued vaccination while highlighting the importance of personalized risk assessment, clinical vigilance for thyroid manifestations, and targeted research to enhance our understanding of immune-endocrine interactions following vaccination.

## Data Availability

All data are available upon request.

## Research registration unique identifying number (UIN)

Registry used: PROSPERO Unique identifying number of registration ID: CRD42024586890 Link: https://www.crd.york.ac.uk/PROSPERO/view/CRD42024586890
